# Revisiting selected ethical aspects of current clinical in vitro fertilization (IVF) practice

**DOI:** 10.1007/s10815-022-02439-7

**Published:** 2022-02-22

**Authors:** Anja von Schondorf-Gleicher, Lyka Mochizuki, Raoul Orvieto, Pasquale Patrizio, Arthur S. Caplan, Norbert Gleicher

**Affiliations:** 1 The Center for Human Reproduction, New York, NY 10021 USA; 2 Chaim Sheba Medical Center, Infertility and IVF Unit, Department of Obstetrics and Gynecology, Sackler Medical Faculty, Tel Aviv University, Tel-Aviv, Israel; 3 Department of Obstetrics, Gynecology and Reproductive Sciences, Miller School of Medicine, University of Miami, Miami, FL USA; 4 Division of Medical Ethics, New York University Langone Medical Center and NYU Grossman School of Medicine, New York, NY USA; 5 Stem Cell Biology and Molecular Embryology Laboratory, The Rockefeller University, New York, NY USA; 6 Foundation for Reproductive Medicine, New York, NY USA; 7 Department of Obstetrics and Gynecology, Vienna Medical School, Vienna, Austria

**Keywords:** In vitro fertilization (IVF), Cryopreservation, Gametes, Oocytes, Embryos, Ethics, Fertility preservation, Egg freezing, Preimplantation genetic testing for aneuploidy (PGT-a), Add-ons to IVF

## Abstract

Ethical considerations are central to all medicine though, likely, nowhere more essential than in the practice of reproductive endocrinology and infertility. Through in vitro fertilization (IVF), this is the only field in medicine involved in creating human life. IVF has, indeed, so far led to close to 10 million births worldwide. Yet, relating to substantial changes in clinical practice of IVF, the medical literature has remained surprisingly quiet over the last two decades. Major changes especially since 2010, however, call for an updated commentary. Three key changes deserve special notice: Starting out as a strictly medical service, IVF in recent years, in efforts to expand female reproductive lifespans in a process given the term “planned” oocyte cryopreservation, increasingly became more socially motivated. The IVF field also increasingly underwent industrialization and commoditization by outside financial interests. Finally, at least partially driven by industrialization and commoditization, so-called add-ons, the term describing mostly unvalidated tests and procedures added to IVF since 2010, have been held responsible for worldwide declines in fresh, non-donor live birthrates after IVF, to levels not seen since the mid-1990s. We here, therefore, do *not* offer a review of bioethical considerations regarding IVF as a fertility treatment, but attempt to point out ethical issues that arose because of major recent changes in clinical IVF practice.

## Introduction

Especially since 2010, in vitro fertilization (IVF) has undergone substantial changes [1], many remaining controversial and raising ethical concerns. Though the ethics of IVF have received considerable attention in the bioethics literature, the clinical infertility literature has paid surprisingly little attention to ethical consequences of recent changes in IVF practice. Here presented review, therefore, concentrates on those clinical changes and their ethical consequences.

### Introductory history

As of 2018, over 8 million world citizens were products of successful in vitro fertilization (IVF) [https://www.eshre.eu/Annual-Meeting/Barcelona-2018/ESHRE-2018-Press-releases/De-Geyter]. By now the number must approach 10 million, establishing IVF as one of the most consequential medical treatments ever introduced. As IVF faced discrimination since its inception, it took 22 years for the Nobel Prize in Medicine and Physiology to be awarded for this accomplishment [2]. In the USA, this discrimination is exemplified to this day by exclusion of IVF from coverage by federal health insurance programs and federal research funding.

Stigmatization may in part derive from the term in vitro* fertilization* itself, describing union of egg and sperm outside of the female body, which, for much of the public, still reflects the beginning of life. Because most human embryos are never given the chance to implant in vivo or in vitro, IVF pioneer Howard W. Jones, Jr., suggested that the term “embryo” be reserved only for embryos with real pregnancy potential. All other embryos he suggested should be called “pre-zygotes” or “pre-embryos” [3]. His argument was that awareness of nature’s wastefulness with human pre-embryos might make it obvious that fertilization could not reflect the beginning of human life. How discriminatory the maternal endometrium behaves in allowing implantation [4] further supports his contention.

Discrimination even involved academia, in pre-IVF days amply demonstrated by the Chairman of Columbia University’s Ob/Gyn department (himself a well-respected fertility specialist) in a court statement, when explaining why he destroyed what likely was the world’s first human IVF experiment, “*because he feared the creation of human monsters*.” [https://www.nytimes.com/2003/02/16/nyregion/dr-l-b-shettles-93-pioneer-in-human-fertility.html].

“Frankenstein” analogies continued permeating media commentaries [5]. As recently as 2010, a German medical publication still described Bob Edward’s Nobel prize as “*rewarding the devil’s work*” [6].

### Why ethics especially matter when it comes to IVF practice

Merriam-Webster defines ethics as the discipline dealing with what is good and bad and with moral duty and obligation [https://www.merriam-webster.com/dictionary/ethic]. Professionally, it is a branch of philosophy, also called moral philosophy that guides what is widely perceived as right or wrong behavior. Ethics, as applied to medicine, is a set of principles to which professionals can refer when making difficult decisions. These principles include respect for autonomy, non-maleficence, beneficence, and justice. Medical ethics weighs each of these values for each of the individuals affected by an action and seeks to achieve the greatest benefit with the least harms. As such, medical ethics differ from religious codes of conduct or political points of view.

What individuals and/or society perceive as right or wrong behavior can differ and is greatly influenced by culture, religion and other factors that have guided infertility-related research and clinical practice to this point [7] and will do so also in the future [8]. Considering that IVF involves production, storage, decisions regarding use or nonuse of embryos as well as decisions regarding their disposal, changes in clinical IVF practice, of course, should include ethical reviews.

## Method of review of literature

We here reviewed the English literature between 2010 and 2021 under key phrases that related IVF (and assisted reproductive technology, ART) to ethics. Because such articles in the medical literature were found to be surprisingly sparse, we also expanded the review to publications in the bioethics literature but only if those articles addressed changes in IVF practice. So, for example, we reviewed articles regarding preimplantation genetic testing of embryos and regarding so-called social or planned egg-freezing since both subjects were substantially affected by changes in IVF practice over recent years. We, however, did not include the substantial literature on the permissibility of sex selection since this issue, while ethically a very relevant subject, was not affected by changes in IVF practice since especially 2010. In addition, we also searched publications in lay media if there was reason to believe that they may offer additional information.

Medical and bioethical literatures were searched through PubMed, Medline, and Google Scholar, though not through PhilPapers; the lay press was searched through Google search. Search phrases were broad and included < ethics of IVF > , < ethics of assisted reproduction/ART > , < ethics of egg/oocyte freezing/ cryopreservation > , < ethics of embryo freezing/cryopreservation > , < ethics of reproductive tissue freezing/ cryopreservation > , etc. Positions statements of professional societies were specifically queried by searching their policy statements, opinions and/or published guidelines. Attitudes toward different causes of embryo losses were, similarly, searched with appropriate key words. Where information was obtained from the Internet, relevant addresses are noted in the text.

**Fig. 1:**
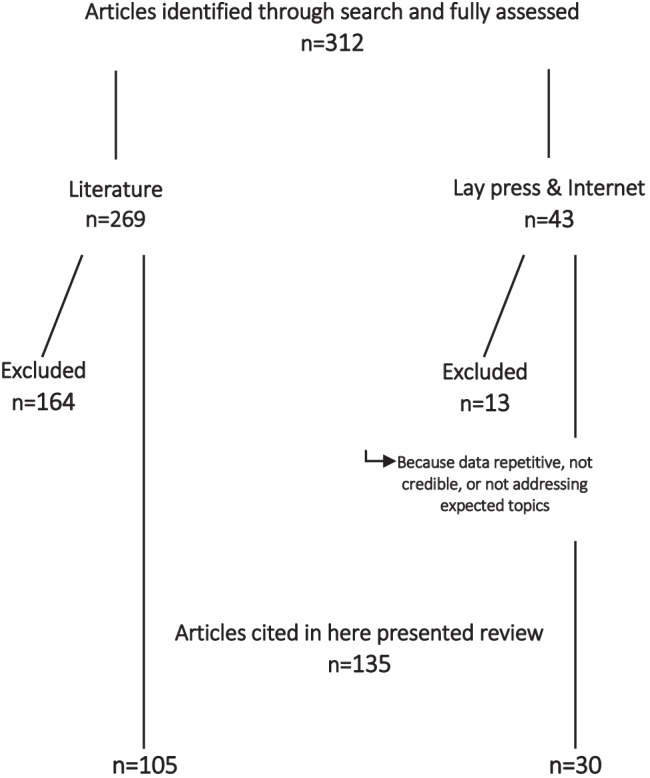
Flowchart of literature review

We reviewed a total of 312 articles in medical literature and lay press combined. Among those, 105 are cited as references and 30 as electronic addresses from lay press and Internet (Fig. 1). Through those we identified six mega-themes in which changes in IVF practice called for an ethical reassessment: (i) Are human embryos deserving of special consideration?; (ii) ethical responsibilities of professional organizations; (iii) ethical responsibilities of physicians and IVF centers; (iv) consequences of commercialization of IVF; (v) data reporting in the literature; and (vi) cryopreservation of gametes, embryos, and reproductive tissues. They are addressed in order in the next section of this manuscript.

This manuscript did not have predetermined research questions beyond, “*what ethical considerations must be considered based on substantial changes in IVF practice*,” especially since 2010. The manuscript, therefore, cannot be considered a systemic review, and selection of here presented six mega-themes may be biased. This review also did not include a formal assessment of quality of evidence. Such assessments are difficult to reach in discussing ethical issues, where opinions may significantly differ.

## Results of literature review

### Are human embryos deserving of special considerations?

Ethicists disagree whether human embryos deserve special considerations [9–11]. So-called artificial or synthetic embryos, produced from stem cells, have also become subjects of debate [12, 13], with some ethicists expressing concern that they could develop consciousness [14].

Recent *ASRM* (*American Society for Reproductive Medicine*) guidelines, therefore, were timely [15] when supporting human embryo research. Such research must, however, be: (i) pre-approved by an Institutional Review Board (IRB); (ii) scientifically substantial enough to warrant use of human embryos; (iii) knowledge should not be obtainable by alternative non-human model systems; (iv) must be conscious of scarcity of human embryos donated to research; and therefore, (v) use only the smallest numbers of embryos required for an experiment.

Special considerations for human embryos have also been codified in the so-called 14-day rule, stipulating that for research purposes embryos cannot be grown in laboratories beyond 14 days from fertilization. The rule arose out of 1979 reports in the USA and UK from, respectively, the *Ethics Advisory Board to the U.S. Department of Health, Education and Welfare (HEW)* [https://repository.library.georgetown.edu/bitstream/handle/10822/559350/HEW_IVF_report.pdf] and *the U.K. Warnock Committee of Inquiry Human Fertilisation and Embryology* [https://www.bakerinstitute.org/media/files/files/8a0b4eac/chb-pub-greenwall-intl-012219.pdf]. Now regarded by some researchers as outdated [16, 17], it may be on the verge of being expanded [8, 18, 19].

Because IVF maintains embryos only up to day-7 after fertilization, the “14-day rule” is not relevant for clinical IVF. But since IVF now produces human embryos on an almost industrial scale, how they are selected or deselected, cryopreserved or freshly transferred and why and/or how embryos may be disposed of are, all, issues deserving an updated ethical review. Of special concern are also embryos “abandoned” by their owners, piling up in IVF centers [20].

### Ethical responsibilities of professional organizations

Guidance of ethical clinical practice is a core function of professional organizations. Federal funding restrictions for reproductive medicine, however, exposed vulnerabilities of organizations in the field which made them unduly dependent on industrial funding sources and, at times, created the impressions of conflicts of interest. A most telling example is preimplantation genetic testing for aneuploidy (PGT-A), in the mid-1990 first called preimplantation genetic screening (PGS). Though having issued two guidance documents in 2008 and 2018 clearly stating that PGS/PGT-A failed to demonstrate promised outcome benefits for IVF [21, 22], the *ASRM* to this day failed to comment on the rapidly expanding clinical utilization of the procedure in the U.S.

Demands for restricting PGT-A use to experimental protocols had to, instead, come from individuals [23–25] and small, spontaneously coalesced groups [26]. *ACOG’s* (*American College of Obstetricians and Gynecologists*) recent Committee Opinion also only acknowledged limitations of PGT-A [27], while *ESHRE (European Society for Human Reproduction and Embryology)* in a series of opinions came surprisingly close to endorsing PGT-A as an “established” procedure [28–30]. Prominent experts in the field to this day advocate for the procedure [31]. Though associated with worldwide declining pregnancy and live birth rates [32], other “add-ons” to IVF (Table 1) have also only received limited attention from professional organizations [33, 34]. In contrast, neither ethicists [35] nor individual professionals [36, 37] have hesitated to point out how unethical utilization of “add-ons” is in absence of clinical benefits. Among authoritative bodies, only the *British Human Fertilisation & Embryology Authority (BHFEA),* a quasi*-*government-organization, addressed the issue in a practical intervention by establishing a “traffic light rating” for “add-ons” [https://www.hfea.gov.uk/treatments/treatment-add-ons/].

**Table 1:** Selective newly added practices to IVF considered “add-ons”

Strongly associated with declining live birth rates in fresh non-donor IVF cycles
• Preimplantation genetic testing for aneuploidy (PGT-A) • Universal extended culture to blastocyst stage* • Universal single embryo transfer • Mild ovarian stimulation • Embryo banking with delayed transfer
Secondary “add-ons” with smaller or no obvious adverse effects on IVF outcomes
• Endometrial injury/scratching • Physiological intracytoplasmic sperm injection (PICSI) • Intracytoplasmic morphologic sperm injection (IMSI) • Receptivity testing • Universal intracytoplasmic sperm injection (ICSI) • Artificial egg activation • Embryo glue • Immune suppression treatments

^*^ In good prognosis patients, blastocyst-stage culture offers a mild shortening in time to conception and, therefore, should not be considered an “add-on.”

Economists recently also offered an important argument against the increasing utilization of “add-ons:” As costs for “add-ons” in the marketplace greatly vary, the economic argument can be made that they simply have no objective economic value. [38].

### Ethical responsibilities of physicians and IVF centers

Four ethical principles, beneficence, non-maleficence, respect for patient autonomy and justice, are supposed to direct medical practice [39]. Reproductive medicine, however, often faces additional socio-legal conditions for practice that can significantly differ between countries and even between US states, though government interventions into IVF practice have not been too successful. For example, restrictions on the use of third-party donor eggs or gestational carriers only increased medical tourism [40]. Attempts to reduce multiple pregnancies by Canadian and European governments by financially incentivizing elective single embryo transfers (eSETs) were well-meaning but, ultimately, also failed [41–44].

They also appear ethically questionable since they can be viewed as restricting patients’ rights to self-determination. Women with long-standing infertility, for example, are often, understandably, desirous of twin pregnancies [45]. Their logical desires, therefore, deserve at least serious consideration [46]. The increasingly widespread opinion in fertility practice that all twin pregnancies must be viewed as “adverse outcomes” [47], surprisingly also supported by guidance from some professional organizations, must therefore be challenged on ethical grounds.

### Ethical consequences of the commercialization of ART

#### Industrialization and commoditization of IVF

**Fig. 2:**
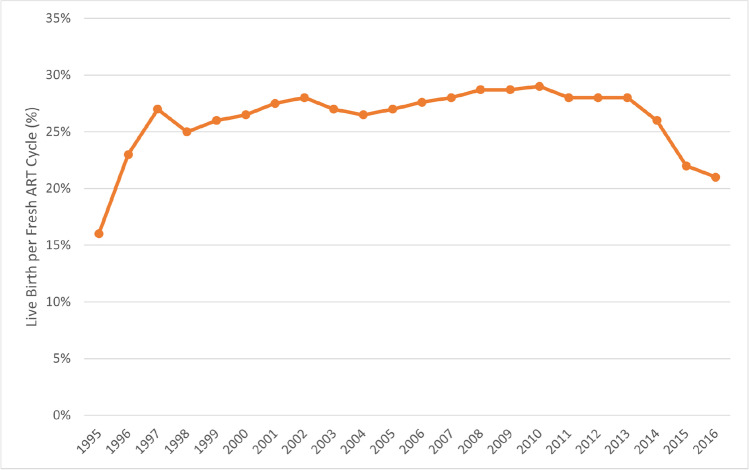
US non-donor fresh live birth rates in autologous IVF cycles 1995–2016. This figure is with permission modified from Gleicher et al. [32] and is based on the Annual CDC ART Success Rate Reports in the years 1995–2016. Non-donor fresh livebirth rates demonstrated almost steady improvements until 2010. An initial mild decline till 2013 between 2013 and 2016 turned into a very profound decline by 2016 to levels not seen since the mid-1990s

The introduction of vaginal egg retrieval under ultrasound control [48] freed IVF from being a hospital-based procedure and brought it into small ambulatory settings which competed mostly based on their IVF cycle outcomes. Large and geographically dominant IVF centers first evolved in states with early legislative insurance mandates for IVF coverage, like Massachusetts and Illinois, and can now be viewed as the forerunners for industrialization and commoditization of IVF practice. By medical insurance companies through their provider networks now determining patient-flows, the competition between IVF clinics radically changed from emphasis on cycle outcomes to competition in reimbursement rates offered to insurance providers. Lower reimbursement rates, in turn, mandated new administrative discipline in cost-control, leading in the mid-1990s to first attempts at consolidation of IVF centers. Though US and worldwide IVF cycle outcomes continued to improve until approximately 2010, these changes have characterized IVF ever since, ultimately resulting in steady declines in clinical pregnancy and live birth rates since 2010 (Fig. 2), rising IVF cycle costs and declining patient satisfaction in parallel to consolidation, industrialization, and commoditization [32]. As in prior publications, we here define “industrialization” as change from a private practice model to corporate/investor ownership of IVF practice, usually accompanied by “commoditization,” defined by prioritization of revenue and profit over IVF outcomes.

For example, only three corporations control three-quarters of the IVF market in Australia and New Zealand [https://www.ibisworld.com/au/market-size/fertility-clinics/], as investors “*see big money in infertility*” [https://www.statnews.com/2017/12/04/infertility-industry-investment/], and private equity in Europe went on a buying spree of IVF clinics. In the USA, venture capital now controls the largest clinic network of IVF providers in the nation [https://www.institutionalinvestor.com/article/b1ff3x6hcl5wbb/This-Venture-Capital-Fund-Wants-to-Get-You-Pregnant] and institutional capital owns at least one-third of U.S. IVF cycles. The industrialization of IVF as of this point, therefore, appears unstoppable and raises serious practical concerns, but also many concerns of ethical nature.

Once again, Australia is a good example: The country’s IVF live birth rates declined in parallel to increasing industrialization and corporate control of IVF practice, while cycle costs increased and patient satisfaction declined [32]. A recent class action suit appears reflective of these developments: Over 100 patients accused Monash IVF, one of Australia’s leading IVF providers, of inadvertently destroying healthy embryos based on noninvasive PGT-A (niPGTA) and plaintiffs are expected to increase tenfold [https://www.abc.net.au/news/2020-12-23/class-action-against-monash-ivf-fertility-clinic/13010682].

niPGT-A is the fourth and most recent incarnation of PGT-A, likely the most consequential “add-on” to IVF, which to this day established no outcome benefits for IVF and, indeed, likely adversely affects live birth chances for many women [25, 49].

Likely mistakenly, damage from embryo biopsies has been assumed by some to be the primary reason for the clinical ineffectiveness of PGT-A [50]. With niPGT-A, fetal DNA could be obtained from embryos’ spent media, eliminating the need for trophectoderm biopsies [51]. In almost all so-far published studies, niPGT-A, however, proved less accurate than traditional PGT-A. A single study supportive of niPGT-A [52] had significant technical shortcomings [53]. Above-noted Australian class action suit claimed niPGT-A to be unreliable in determining whether an embryo is chromosomal-normal or not, leading to false-positive diagnoses of normal embryos and their mistaken discarding, thereby depriving patients of pregnancy and live birth chances [https://www.abc.net.au/news/2020-12-23/class-action-against-monash-ivf-fertility-clinic/13010682].

Considering increasing evidence that utilization of PGT-A outside of experimental protocols appears inappropriate [23, 26, 32, 49–51, 54], similar lawsuits may arise elsewhere. Yet at least three IVF providers in the USA [https://www.ccrmivf.com/news-event/non-invasive-pgt/; https://www.prnewswire.comnews-release/new-hope-fertility-center-open-state-of-the-art-laboratory-300865417.html; http://www.mainlinefertility.com/noninvasivepreimplantation-genetic-testing/] and one national PGT-A laboratory [https://www.prnewswire.com/news-release/non-invasive-pgt-a-launched-clinically-in-usa-300830134.html] already offer niPGT-A as a commercial product.

Euploid births following transfers of embryos by PGT-A labelled as chromosomal-abnormal and, therefore, widely considered “untransferable,” have, moreover, challenged the PGS/PGT-A hypothesis as a whole [55], as has evidence that embryos can self-correct downstream from blastocyst stage [56], thereby eliminating any rationale for embryo testing upstream, whether by biopsy or by noninvasive means.

Growing worldwide PGT-A utilization, therefore, raises several serious ethical concerns, starting with thousands of chromosomal normal human embryos being discarded daily or, under best of all circumstances, just refused transfer. Moreover, IVF clinics often use the alleged unavailability of euploid embryos after PGT-A as an argument to advance patients prematurely into third-party egg donation.

#### Why infertility practice faces special ethical considerations

Whether fee-for-service medical care can under any circumstance be ethical has been in dispute for decades [57]. Ethical complexities in reproductive medicine, however, go beyond financial considerations. National US IVF data from the *Centers for Disease Control and Prevention (CDC)* (https://www.cdc.gov/art/artdata/index.html) and the *Society for Assisted Reproductive Technology (SART)* (https://www.sart.org/patients/a-patients-guide-to-assisted-reproductive-technology/general-information/success-rates/), demonstrate that women after 42–43 are only rarely offered use of autologous oocytes, raising further ethical concerns about age discrimination and the right of self-determination. Physician advice to proceed with third-party donor eggs, based on patient interviews, are often more demanding than recommending (Lyka Mochizuki, personal communication), raising questions whether informed consents are obtained following ethically and legally correct procedure. Similar concerns about proper informed consent also arise when IVF centers mandated PGT-A in every IVF cycle and refuse IVF cycles to patients who do not consent to PGT-A.

Almost evenly split as fees to IVF centers (for embryo biopsy) and genetic testing laboratories (for NGS testing), PGT-A in the USA adds approximately US$ 5,000 for patients in out-of-pocket costs to every IVF cycle. Those costs remain out-of-pocket because the procedure is universally considered an unvalidated experimental procedure and, therefore, not a covered insurance benefit.

#### From medical treatment to social phenomenon

At the onset primarily only a treatment for obstructed or absent fallopian tubes, IVF quickly became a universal treatment for female as well as male infertility. Vitrification in place of slow freezing improved cryopreservation [58] and expanded IVF from a strictly medical procedure into elective-social uses. Those involved newly formatted commercial enterprises, including frozen donor-egg banks [59] and egg-freezing centers, the latter marketing to women to promote longer reproductive lifespans, a process previously described by the phrase “social” and now “planned” egg-freezing [https://www.nbcnews.com/health/features/egg-freezing-startups-have-wall-street-talking-traditional-fertility-doctors-n978526]. These terms were chosen to differentiate egg-freezing for the purpose of extending reproductive lives of women, from the phrase “medical” egg-freezing, which denotes cryopreservation of oocytes (or ovarian tissue) before planned iatrogenic loss of ovarian function, mostly from pending toxic treatments to ovaries, since 2013 no longer considered an experimental treatment by the *ASRM* [59]. The *ASRM,* however, in 2013 specifically declined this designation “*for the sole purpose of circumventing reproductive aging in healthy women*,” at the time called “social” egg-freezing [60]. The cited reasons were insufficient data on safety, efficacy, ethics, emotional risks, and cost-effectiveness.

“Social” egg-freezing, however, continued quick gains in popularity, not the least because of rather blatant misrepresentations by egg-freezing centers that *ASRM’s* above noted declaration regarding “medical” egg-freezing also applied to “social” egg-freezing. By 2018 the *ASRM*’s position inexplicitly changed [61], since no significant new evidence about efficacy of “social” egg-freezing had been developed. In an unprecedented switch, reconsideration of the matter was moved from the Practice Committee (where the earlier 2013 decision had been reached) to the Ethics Committee of the *ASRM*, which, never-before and never since, has addressed clinical practice guidelines. The Ethics Committee’s rationale for the new decision was, “*further reassuring research on efficacy, increasing numbers of women seeking planned oocyte cryopreservation and increasing numbers of physicians providing it.*” [61].

The egg-freezing industry, moreover, moved into truly dystopic territory: One company featured in a cover story in Forbes Magazine, for example, announced a business plan to revolutionize future reproduction by freezing female and male gametes at young ages (i.e., peak fertility), so, when ready to reproduce, couples could use these at younger ages cryopreserved gametes to produce embryos, test those by PGT-A and transfer only “euploid” embryos [62]. The company raised U.S. $200 million in initial financing for this asexual futuristic reproduction model.

Heavily promoted by investor-owned clinic networks, initial media support for “planned” egg-freezing was glowing. Not even “egg-freezing parties” for young women that served alcohol were considered out of line in those days [https://www.theguardian.com/science/2018/jan/02/egg-freezing-parties-wall-st-fertility-women; https://www.chicagotribune.com/lifestyles/health/ct-egg-freezing-parties-20171116-story.html].

The bubble, however, did not last. “Planned” egg-freezing demand declined and some newly created egg-freezing chains shut down or had to recalibrate marketing efforts toward more general IVF practice. Ethically, this episode, however, tarnishes IVF practice to this day, as at times misleading promotional efforts for “planned” egg-freezing have changed the perception of IVF practice, increasingly moving it from being a strictly medical practice that remedied disease into a more socially driven practice, akin to cosmetic plastic surgery.

### The ethics of data reporting

Dysfunctional peer review is not unique to the practice of reproductive medicine and does not automatically represent ethical breaches. Through *CDC* and *ASRM/SART* registries, the USA maintains the most comprehensive IVF data reporting system in the world; yet, because of excessive access restrictions, formal analyses of those data by independent investigators are sparse. That worldwide IVF cycle outcomes in fresh non-donor cycles from 2010 on started declining went, until recently, therefore, unnoticed [32] (Fig. 2). As noted earlier, substantial responsibility for these declines appears to lie with “add-ons” to IVF.

Publications of unvalidated outcome claims and failing to police correct data reporting that differentiates between concepts and ideas (i.e., a hypotheses) and validated evidence, makes at times a dysfunctional peer-review process share in the responsibility for these developments [63, 64]. Errors in peer review are then often further augmented by the habit of editors to ask reviewers who contributed poor peer reviews to write accompanying commentaries. The so-created vicious circles then promoted unvalidated treatments in routine clinical IVF practice.

The quality of peer review has in general suffered over the last decade, and ethical missteps increased even in prominent journals [63–67], and https://ssrn.com/abstract=2946811 or https://dx.doi.org/10.2139/ssrn.2946811]. A major cause has been the explosive growth in new journals and submitted manuscripts and the inability to find good peer reviewers for increasing numbers of submissions. Increasing tolerance of potential conflicts of interest and the politicization of editorial offices, however, are, self-inflicted wounds. Medical journals are not meant to be places of political discourse [68]. Their primary purpose is to serve scientific discourse.

#### Conflicts of interest in peer review

Conflicts of interest in editorial offices, unfortunately, still abound [66, 67, 69]. Concomitantly, opinions questioning powerful commercial interests are often censored. One, for example, must ask, why else prominent investigators would publish a paper critical of universal blastocyst-stage culture in an ultrasound journal [70]. Similarly, several important papers opposing PGT-A utilization had to resort to publication in general medical journals [24, 49], as PGT-A-related peer review in prominent reproductive medicine journals until recently was practically controlled by proponents of the procedure.

#### Patient selection biases

The ultimate responsibility for ethical data presentation lies, however, with authors. Biased patient selection is a good example, often aggravated by extrapolation of outcomes in favorable-to less-favorable patients. One of the most blatant examples is extended embryo culture to blastocyst stage, nowadays a routine practice in most IVF centers. Outcome advantages from extended embryo culture are, however, only marginal, have been demonstrated only in good prognosis patients [71] and appear outweighed by increased neonatal morbidity [70, 72]. A recent Australian study suggested that in unselected patients, cleavage-stage transfers significantly outperform blastocyst-stage transfers [73].

Routine culture to blastocyst-stage can be viewed as the first “add-on” to IVF with adverse effects on worldwide live birth rates. Alleged benefits of almost all IVF “add-ons” were initially reported in highly selected good-prognosis patients.

#### Utilization of incorrect statistical methodologies

IVF cycle outcomes must not be reported with reference embryo transfer. As such reporting also selects out good-prognosis patients, it again is misleading. The correct reference point for analysis, therefore, must be cycle start (“intent to treat”). Yet, some of the most frequently cited studies in IVF practice, including prospectively randomized studies frequently referred to in false claims for “add-ons,” either used embryo transfer as reference point and/or incorrectly randomized patients at embryo transfer. The PGT-A procedure is again a good example for this misleading practice [74] and, considering how often this issue has been addressed by now in the literature, continuing the practice and/or extrapolating outcomes from highly selected patients to the general population must be considered ethically unacceptable intended transgressions.

With even physics, the science most rigorously demanding statistically irrefutable evidence, at times affected by methodical errors in analyses [75], authors, editors, and publishers of scientific journals conjointly share in the responsibility of discovering such errors before publication. Infertile patient populations represent on average approximately 15% good-, 70% average-, and 15% poor-prognosis patients [76]. Treatments only observed in good-prognosis patients, therefore, will benefit only roughly 15% of patients. That the remaining 85% demonstrate either no benefit or may even be harmed must be a key consideration in every peer review.

### Ethics of cryopreservation of gametes, embryos, and reproductive tissue

The first organization to formally approve ovum donation and embryo freezing was the U.K.’s *Royal College of Obstetricians and Gynecologists* in 1983 [77]. Due to HIV, in the USA, a freewheeling practice of fresh donor-semen inseminations came to a sudden halt in the mid-1980s, and donor-semen cryopreservation with mandated 6-months quarantine became standard of care [78]. In parallel, cryopreservation of human embryos became routine. A first pregnancy (twins) following cryopreservation and thawing of oocytes was reported in 1986 [79], but cryopreservation was not common practice until 2005, when vitrification started replacing slow freezing. At roughly the same time, “medical” fertility preservation through cryopreservation of ovarian tissue evolved primarily for young women with cancer and other diseases requiring ovary-toxic treatments [80].

**Fig. 3:**
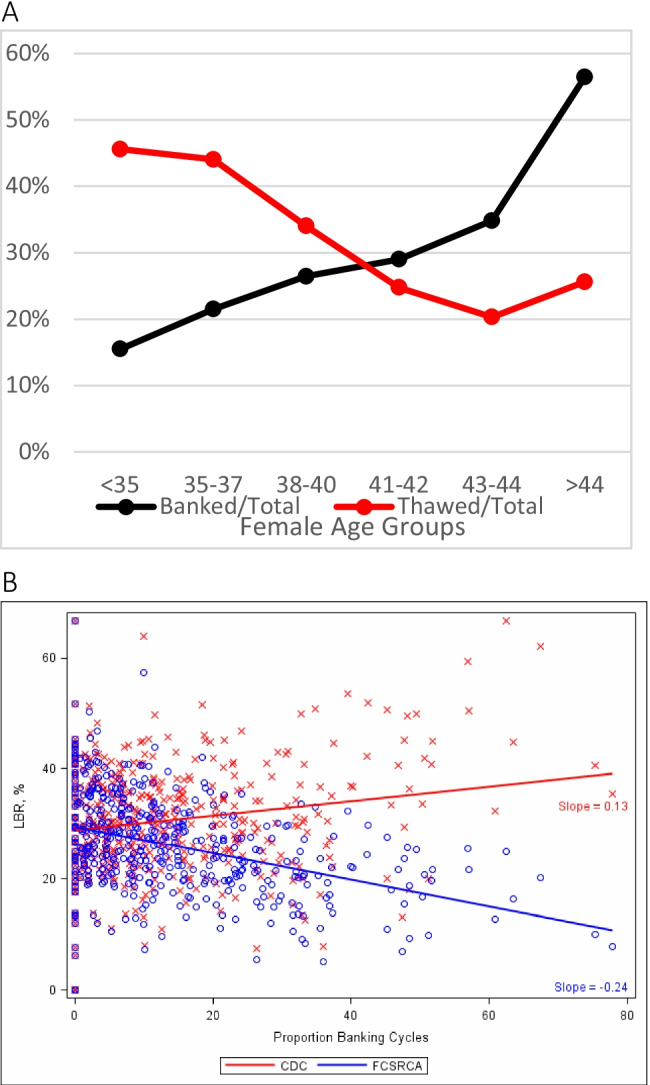
Embryo banking and thawing in the USA by age of patient. **A: US IVF centers reporting to the CDC** in 2013 modified with permission from Kushnir et al. [92, 93]. It depicts growing number of “banked” cycles with advancing female age, yet declining numbers of thawed cycles and, therefore, significant patient selection biases by US IVF centers in embryo cryopreservation disfavoring older women by not even using in older women embryos even in thaw cycles. Thereby not completing cycles with embryo transfers, those cycles’ outcomes then did not appear in the center’s outcome statistics reported to CDC. By removing older patients from consideration, better prognosis patients are left, artificially improving a center’s IVF cycle outcomes, demonstrated in **B: Live birth rate of banked cycles** according to two different calculations: The red line reflects live birth rates under the at that time reporting guidelines to the CDC that allowed exclusion of unresolved cycles and clearly suggests outcome improvements with higher banking rates. The blue line, in contrast, excluded banking cycles, demonstrating that rates actually declined with increasing banking. The authors also reported that only 13/341 IVF centers (3.8%) accounted for 50% of all from CDC-reporting excluded cycles. They uniformly were among the US centers reporting highest live birth rates. Once cycles were, however, appropriately adjusted, these centers’ live birth rates equally uniformly fell below the median of remaining over 300 centers

Cryopreservation grew rapidly because of “planned” egg-freezing, further enhanced by growth in donor egg banks and a surge in utilization of frozen donor oocytes, rapidly supplanting fresh donor cycles [81]. In parallel, “embryo banking,” however, flourished with routine IVF (Fig. 3), which must be attributed to yet another unvalidated hypothesis. Like most unvalidated “add-ons” to IVF, the hypothesis that ovarian hyperstimulation creates an unfavorable hormonal transfer milieu in IVF that could be improved if transfers were to be delayed to a subsequent frozen-thawed cycles [82, 83], has since been convincingly refuted [84]. Nevertheless, embryo banking is, still, widely practiced.

#### What ethicists have to say

In contrast to other IVF-related clinical practice changes, the growth in “social,” now called “planned” egg-freezing, has attracted significant commentary in the literature from ethicists. Quoting a few, Bhatia and Campo-Engelstein commented on how *ASRM* and *ESHRE*, independently, already in 2012 in diverging opinions propelled commercial markets in “social” egg freezing [85]: Claiming to support reproductive autonomy and justice, *ESHRE* approved of the process, while *ASRM* at that point still discouraged it, though individual ethicists were supportive [86]. Supported by media that lavishly lauded several high-tech companies for offering female employees (only very limited) financial support for “social” egg-freezing, commercial markets in the USA chose to mostly ignore the *ASRM*’s guidelines. A more balanced view, however, ultimately prevailed [87] and ethicists, indeed, started wondering whether company-sponsored egg freezing promotes, or confines, women’s reproductive autonomy. A majority concluded that reproductive autonomy was not served by offers of financial support for egg banking [88].

Claudia Bozzaro recently investigated whether “social” egg freezing was “*a good response to socioeconomic and sociocultural constraints, leading women to postpone motherhood*,” allowing them “*a flourishing life*,” as known in virtue ethics. She, too, concluded in the negative, that “social” egg freezing cannot be viewed as an adequate response to cultural factors since those cannot be resolved by simply extending a woman’s fertility [89]. Based on life phases and related normative expectations, Eva Weber-Guskar was more positive: She concluded that nothing really speaks against using “social” egg freezing, though she also offered certain limitations to prevent abuses [90].

Professionals as well as lay media have since become more cautious in commiserating about “planned” egg-freezing. Goldman summarized uncertainties by noting that many (if not most) patients will not achieve pregnancy with cryopreserved oocytes. She, therefore, stressed the ethical importance of providing patients with correct information [91]. A most stinging rebuke came, however, from a clinical psychologist and associate professor at the *Feinberg School of Medicine of Northwestern University* who, in an unfortunately since removed posting under the title, “*Glamorizing egg freezing can have devastating consequences*” noted that, “*medical and social communities have misled women for far too long about the realistic chances of getting pregnant*.”

#### Unintended consequences

Every aspect of medicine can be affected by unintended consequences. As Japan’s IVF experience demonstrates better than that of any other country, regional IVF markets compensate for losses in live birth rates almost one-to-one with more cycle starts. Losing over a decade approximately two-thirds of live births by adopting a mild-stimulation protocol with mandated blastocyst-stage culture as the nation’s primary protocol, the country in parallel tripled IVF cycle starts [32].

Demonstrating how in the USA embryo banking cycles with advancing patient age counterintuitively steadily increased, while thawed cycles decreased, Fig. 3a demonstrates yet another likely unintended consequence: Preferential cryopreservation of embryos from older women represents just another way to select against poor prognosis patients, thereby helping a center to inflate overall pregnancy rates [32, 92, 93]. This was previously noted based on formal US IVF outcome reporting. When such biased reporting involving especially IVF centers with high percentages of embryo banking cycles was appropriately adjusted by adding back previously excluded banking cycles preferentially applied to older women (Fig. 3B), revised analyses demonstrated in some of the nation’s largest IVF centers significantly lower live IVF birth rates. They, indeed, had overreported live births up to 4.5-fold [92, 93]. Improvements in cryopreservation, thus, were misused to harm mostly older women [94].

#### Selling frozen gametes (and embryos)

Paying semen donors has been considered ethical practice for decades. With establishment of sperm banks, the process was later fully codified. Establishment of frozen egg banks more recently, also finally codified payments to egg donors, and defined oocytes as “products of value.” This is, however, not how, until only several years ago, egg donation was perceived in the U.S.: Practiced since the mid-1980s, egg donors were paid substantial fees. The ethical framework for these payments was, however, disingenuous by pretending payments were made only to reimburse donors “for time and effort.” This charade ended abruptly in April of 2011, when *ASRM* and its daughter society, *SART* (*Society for Assisted Reproductive Technology*), were accused of price fixing in a lawsuit because the societies had issued compensation guidelines for egg donors to U.S. IVF centers [95]. In a settlement, *ASRM* agreed in February of 2016 to withdraw its guidance [https://www.iflg.net/settlement-ends-price-guidelines-for-egg-donor-fees/], thereby acknowledging that egg donation was a commercial transaction. Establishment of commercial donor-oocyte banks at approximately the same time further confirmed eggs as another commercial reproductive product, not different from donor semen.

Many IVF centers have since transitioned from using fresh to primarily utilizing frozen donor eggs. By 2018 frozen donor egg cycles in the USA, indeed, exceeded fresh cycles. Partially, this quick transition was, however, again the consequence of false claims: Though national data from the beginning demonstrated lower pregnancy and live birth rates from frozen eggs [96], the egg-freezing industry incorrectly insisted that outcomes were identical [97]. Recent reports suggest a ca. 12% lower success rate with frozen donor eggs.

New York state prohibits production of human embryos “on-speculation” [https://www.health.ny.gov/regulations/task_force/reports_publications/execsum.htm;]. States without such restrictions, however, allow IVF clinics to produce embryos from donor eggs and donor sperm without predetermined “parents.” Like gametes, those donor-embryos can then be offered in anonymous embryo donations at predetermined per-embryo fees. Human embryos have, thus, also become a tradable commodity, even though ethical guidelines for embryo donation still prohibit the “sale” of embryos [98]. How selling human embryos can ethically coexist with the notion that human embryos are deserving of special consideration remains to be explained.

### Specimen loss

With how much effort cryopreserved embryos are protected during even natural disasters [99] is on the other hand good evidence for the special considerations given to human embryos for ethical but also practical-legal reasons. Specimen loss, nevertheless, occurs and can have several causes.

#### Accidental specimen loss

In March of 2018, due to failures of cryopreservation storage tanks, two prominent IVF centers at almost the same time suffered catastrophic specimen losses [https://www.chicagotribune.com/nation-world/ct-frozen-eggs-embryos-legal-question-20180824-story.html]. Small-scale accidental thaws had been reported in the literature before [100], but they never approximated this scale. *The Washington Post* four months later reported that a preliminary investigation of one of the centers concluded that the loss of 4,000 eggs and embryos from 700 patients was “largely preventable” [https://www.washingtonpost.com/national/health-science/patients-mobilize-to-take-legal-action-against-fertility-clinics-with-malfunctions/2018/03/12/15ff62d6-2633-11e8-bc72-077aa4dab9ef_story.html]. Ethical as well as legal consequences of such an event having been preventable, are, however, profound.

#### Specimen loss by decree

Is another possibility, first pointed out by Nobel laureate Robert G. Edwards, when reporting on the disposal of large numbers of cryopreserved embryos, in the UK then time-limited by government decree [101]. This also is an appropriate description for what currently happens to large numbers of “abandoned” human embryos, by their owners no-longer “wanted”. In the USA such embryos may be ethically disposed but are prohibited from any other use. Prohibition from clinical use appears ethically correct. Their utilization for IRB-approved research is currently, however, also prohibited [102] and more difficult to understand [20, 103]. Rapid accumulation of unwanted frozen oocytes threatens to become the next big maintenance problem for IVF laboratories [104].

A recent *New York Times* article in detail pointed out difficulties in deciding what to do with excessive embryos [https://www.nytimes.com/2020/04/15/parenting/fertility/ivf-unused-frozen-eggs.html]. Patients have in principle three legal options: (i) Their embryos can be anonymously donated through a fertility center to other infertile couples; (ii) embryos can be donated to research; and (iii) IVF clinics can be instructed to “ethically” destroy those embryos. Many patients, however, choose a fourth option, not listed in consent papers signed when embryos are cryopreserved. Their choice was, *not to decide*! By becoming unresponsive to further communications from their IVF clinics, they passed the decision for roughly one-third of all frozen embryos (the percentage of currently abandoned embryos in the U.S.) back to their IVF centers. [https://www.nbcnews.com/health/features/nation-s-fertility-clinics-struggle-growing-number-abandoned-embryos-n1040806] and, therefore, practically, entrusted the fate of their obviously unwanted embryos to their IVF centers. Those under current ethical guidance and the law, are given, however, only the singular choice of disposal [102].

#### Specimen loss in course of unvetted clinical practice

Only a minority of IVF cycles leads to delivery of offspring. Most failures are unpreventable at current knowledge levels. Some are, however, potentially preventable if they involve medical negligence, including the use of potentially harmful treatments to IVF outcomes. Previously unvalidated “add-on” to IVF, now often routinely offered to the public (or in some cases even mandated by IVF clinics) and causing harm to a patient’s pregnancy chances, are a good example. Likely again the most consequential “add-on” to current IVF practice is PGT-A. Its continuing use reflects an obvious ethical breach of medical practice, considering the nonuse and/or disposal of large numbers of embryos with significant pregnancy potential because of PGT-A [25, 49].

#### Disposition and posthumous use of embryos and gametes

Complex ethical and legal issues may also arise related to disposition of gametes and embryos in the event of divorce or death of one of the prospective parents. Reflecting society’s difficulties in defining the moral, ethical, and legal status of human embryos, such cases can become legal battle grounds [105]. Several court cases have been waged and legislative efforts initiated to change the currently widely held consensus that both partners must agree to use of previously cryopreserved embryos. Demand for posthumous use of cryopreserved gametes and/or embryos has increased. IVF centers, therefore, are facing increasing ethical and legal complexities in the multitude of possibilities that can arise from desires of relatives of deceased individuals to assume ownership of gametes, embryos or reproductive tissues. The Ethics Committee of the *ASRM* recently addressed this issue [61].

## Conclusions

Though not a very popular subject in the clinical infertility literature, we found these identified clinical practice changes in association with IVF to be more complex and interesting in their ethical relevance than initially anticipated. Like most manuscripts, this one has limitations and omissions since ethical issues in reproductive research and clinical practice are almost unlimited. Though ethicists since inception of IVF have steadily opined on important subjects relating to IVF, they have done so mostly relating to “big questions,” like the 14-day rule or production of artificial embryos for research purposes; what this review confirmed to be sparse is a steady eye of ethicists upon practice changes in association with IVF that may appear minor when introduced but, as hopefully here well demonstrated, can have highly significant ethical consequences for the field. What the field experienced over the last decade in clinic and research laboratories and where activity likely will go to in the coming decade, was recently summarized in two manuscripts readers are referred to for further insights [7, 8].

**Support:** This study was supported by intramural funds of the CHR and the Foundation for Reproductive Medicine.
